# Identification of lncRNAs associated with the progression of acute lymphoblastic leukemia using a competing endogenous RNAs network

**DOI:** 10.32604/or.2022.027904

**Published:** 2023-02-09

**Authors:** SHAHRAM NEKOEIAN, TAHEREH ROSTAMI, AMIR NOROUZY, SAFIN HUSSEIN, GHOLAMREZA TAVOOSIDANA, BAHRAM CHAHARDOULI, SHAHRBANO ROSTAMI, YAZDAN ASGARI, ZAHRA AZIZI

**Affiliations:** 1 Department of Molecular Medicine, School of Advanced Technologies in Medicine, Tehran University of Medical Sciences, Tehran, Iran; 2 Research Institute for Oncology, Hematology and Cell Therapy, Shariati Hospital, Tehran University of Medical Sciences, Tehran, Iran; 3 Department of Energy & Environmental Biotechnology, National Institute of Genetic Engineering and Biotechnology (NIGEB), Tehran, Iran; 4 Department of Medical Laboratory Science, University of Raparin, Rania, Kurdistan Region, Iraq; 5 Department of Medical Biotechnology, School of Advanced Technologies in Medicine, Tehran University of Medical Sciences, Tehran, Iran

**Keywords:** RT-qPCR, ALL, Non-coding RNAs, microRNA, Leukemia

## Abstract

Acute lymphoblastic leukemia (ALL) is a malignancy of bone marrow lymphoid precursors. Despite effective treatments, the causes of its progression or recurrence are still unknown. Finding prognostic biomarkers is needed for early diagnosis and more effective treatment. This study was performed to identify long non-coding RNAs (lncRNAs) involved in ALL progression by constructing a competitive endogenous RNA (ceRNA) network. These lncRNAs may serve as potential new biomarkers in the development of ALL. The GSE67684 dataset identified changes in lncRNAs and mRNAs involved in ALL progression. Data from this study were re-analyzed, and probes related to lncRNAs were retrieved. Targetscan, miRTarBase, and miRcode databases were used to identify microRNAs (miRNAs) related to the discovered genes and lncRNAs. The ceRNA network was constructed, and the candidate lncRNAs were selected. Finally, the results were validated with reverse transcription quantitative real-time PCR (RT-qPCR). The ceRNA network outcomes demonstrated that the top lncRNAs associated with altered mRNAs in ALL are IRF1-AS1, MCM3AP-AS1, TRAF3IP2-AS1, HOTAIRM1, CRNDE, and TUG1. Investigations of the subnets linked to MCM3AP-AS1, TRAF3IP2-AS1, and IRF1-AS1 indicated that these lncRNAs were considerably related to pathways associated with inflammation, metastasis, and proliferation. Higher expression levels of IRF1-AS1, MCM3AP-AS1, TRAF3IP2-AS1, CRNDE, and TUG1 were found in ALL samples compared to controls. The expression of MCM3AP-AS1, TRAF3IP2-AS1, and IRF1-AS1 is significantly elevated during the progression of ALL, playing an oncogenic role. Due to their role in the main cancer pathways, lncRNAs could be suitable therapeutic and diagnostic targets in ALL.

## Introduction

One of the most prevalent malignancies in children and adolescents is ALL, and substantial research on its related molecular characteristics has been conducted [1,2]. Studies show that the origins of ALL cancer cells are precursors of B and T cells in the bone marrow [3]. Molecular studies in ALL have revealed that mutations, epigenetic alterations, and deregulation of gene expression are all important contributors to the development and malignancy of the disease [4,5]. Despite significant advances in the treatment of ALL, relapse remains a significant therapeutic obstacle. Understanding the molecular mechanisms that contribute to the onset and progression of ALL is, therefore, crucial.

Long non-coding RNAs are a subclass of non-coding RNAs that have been implicated in a variety of biological functions. Extensive research on these RNAs has revealed their potential significance in cancer through modulating pathways including cell proliferation, invasion, migration, metastasis, and major cellular metabolisms [6,7]. Changes in the expression of lncRNAs have been widely reported in various cancers, including ALL. It has been demonstrated that these deregulations can be excellent diagnostic and prognostic targets [8,9]. Moreover, the expression alterations of lncRNAs in the development and malignancy of ALL, as well as their oncogenic and tumor suppressor effects, have been established [10]. Additionally, studies have shown that lncRNAs can affect the post-transcriptional regulation and expression of many genes through interacting and sponging miRNAs. Studies also suggested that lncRNAs contribute to the pathophysiology of ALL by controlling the activity of miRNAs [11,12]. Therefore, lncRNAs can be suitable therapeutic and diagnostic candidates in many diseases, including ALL.

Although extensive studies have been performed on the role and changes of lncRNA expression in ALL, the function and expression of many of them remain unknown. This study aimed to identify lncRNAs that are significantly altered during the development and pathogenesis of ALL through the ceRNA network. For this purpose, a microarray study with access number GSE67684 was analyzed to identify lncRNAs and mRNAs with significant expression changes in ALL. The miRNAs related to lncRNAs and mRNAs were identified using two databases. Next, the ceRNA network was constructed, and the lncRNAs with the highest criteria were selected. Finally, the in-silico results were evaluated using the RT-qPCR technique.

## Materials and Methods

### Differential gene expression analysis

To identify altered lncRNAs and mRNAs in ALL, the raw data of GSE67684 were downloaded from Gene Expression Omnibus (GEO) and processed. Initial data preprocessing such as background correction, data normalization based on the Robust Multi-array Average (RMA) approach, removal of genes with zero expression levels, and data conversion to binary logarithmic form were conducted [13,14]. The data in this study were based on two different platforms, of which only GPL570 (Affymetrix hgu133Plus2) was considered. The study is based on 381 bone marrow samples of ALL patients collected at diagnosis (day zero) and day eight of remission-induction therapy. The start and the end of each gene, their corresponding chromosome bands, and their biological function were retrieved based on Affymetrix HGU133Plus2 identifiers using the Biomart package. Subsequently, the probes associated with lncRNAs and mRNAs were identified [15]. Further, the mean expression of each probe was used to investigate the differentially expressed mRNAs (DEMs) and lncRNAs (DELs) in each group. After normalization, the differences in the expression of all genes, including mRNAs and lncRNAs were evaluated in day eight patients compared to day zero patients. The expression of mRNA and lncRNAs in day eight samples was compared to day zero considering |logFC| > 1 and FDR 0.01.

### Construction of ceRNA network

To identify miRNAs that can potentially interact with DELs, miRcode data were used, and the association between DELs and miRNAs was evaluated [16]. This database contains more than 10,000 lncRNA-miRNA interactions. Targetscan (www.targetscan.org) and miRTarBase (http://miRTarBase.cuhk.edu.cn) datasets databases were also utilized to obtain miRNAs that can bind to DEMs, MicroRNAs validated by both databases were selected for further processing. Furthermore, Overlapping miRNA-mRNA interactions were removed from the analysis process. Subsequently, the identified interactions between miRNA-lncRNA and miRNA-mRNA were used to construct the ceRNA network. Finally, the miRNA-mRNA-lncRNA network was constructed using obtained results from previous steps and plotted by Cytoscape software [17].

### Data enrichment

In order to discover the functions of lncRNAs obtained from the ceRNA network, enrichment analysis was performed using the Molecular Signatures Database (MsigDB) repository from the Enrichr database (maayanlab. cloud/Enrichr). The mRNAs associated with candidate lncRNAs were used for enrichment [18]. Finally, Benjamini-Hochberg corrected *p*-value of less than 0.01 was considered for the enriched pathways.

### Key subnets associated with candidate lncRNAs

To investigate the role and possible pathways in which each identified lncRNA could be involved, subsystem analysis and assessment of DEMs associated with candidate lncRNAs were used. For this purpose, lncRNAs and related miRNA-mRNAs were extracted separately. Then, the subnetworks were constructed and illustrated by Cytoscape software. Finally, the candidate genes associated with each lncRNA were enriched based on the MsigDB repository.

### Sample collection and classification

Blood samples were collected from 33 patients and 15 healthy donors (age range of 10–19 years) referred to the research institute for oncology, hematology, and cell therapy from 2019 to 2021. Samples were then transferred to the laboratory for cytogenetic, flow cytometric, and molecular studies. Based on flow cytometry and molecular findings, all samples were divided into three groups: Philadelphia positive ALL (ph+; N = 9); Philadelphia negative ALL (ph−; N = 17); and T cell ALL (N = 7). Informed consent was obtained from either subjects or legal guardians of minors. The laboratory tests were conducted in accordance with the ethical guidelines and regulations of the Iranian Ministry of Health and Medical Education. This work was approved by the ethical review board with an access number of IR.TUMS.VCR.REC.1399.313. The samples were kept in liquid nitrogen until use.

### RNA extraction, cDNA synthesis, and RT-qPCR

Peripheral blood mononuclear cells (PBMCs) were isolated from whole blood samples using a Ficoll gradient (Sigma Aldrich, Germany) method and according to the manufacturer’s protocol. Total RNA extraction was executed by the TRIzol (Invitrogen, Canada) method. Extracted RNA was treated with DNase (Takara, China) to remove any possible DNA contamination. The AddScript cDNA synthesis kit (AddBio, Korea) was then used to generate cDNA. In order to evaluate the expression of candidate lncRNAs, The RT-qPCR was performed on a 96-well system (AusDiagnostics, Australia) using RealQ Plus 2x Master Mix Green (Ampliqon, Denmark). The sequence of specific primers designed for each lncRNA is summarized in Table 1. The primers were designed using the NCBI blast tool (https://www.ncbi.nlm.nih.gov/tools/primer-blast), and the specificity of each primer was considered. The expression of each gene in each sample was calculated based on 2^−▵Ct^, relative to the internal control GAPDH gene [19].

**TABLE 1:** List of primer pairs used for RT-qPCR with approximate product size

Gene names	5′-3′ sequences	Product length
IRF1-AS1-F	CACACCAAGATCACCTCTG	107 bp
IRF1-AS1-R	ATCCTTTCCTGGTACTGGC	
HOTAIRM1-F	CAATGAAAGATGAACTGGCGA	123 bp
HOTAIRM1-R	CAAACACCCACATTTCAACC	
MCM3AP-AS1-F	GCTGCTAATGGCAACACTGA	119 bp
MCM3AP-AS1-R	AGGTGCTGTCTGGTGGAGAT	
TRAF3IP2-AS1-F	CGTTTTGGCGGCTATGCAG	145 bp
TRAF3IP2-AS1-R	CAGCAGCCATCAAGAATGAGGT	
CRNDE-F	GTTAAGTGTGATGCTTCCAT	134 bp
CRNDE-R	AGCTCCATTATGAATTGCAG	
TUG1-F	TAGCAGTTCCCCAATCCTTG	116 bp
TUG1-R	CACAAATTCCCATCATTCC	
GAPDH-F	GTCTCCTCTGACTTCAACAGCG	131 bp
GAPDH-R	ACCACCCTGTTGCTGTAGCCAA	

### Statistics and software

The R programming language (version 4.1; PBC, Boston, MA, USA) was used to process the GEO dataset, and other analyses (version 4.1). A One-way ANOVA is used to assess statistically significant differences between the means of the two groups. The *t*-test was conducted to identify statistically significant DELs and DEMs using the *p*-value and adjusted *p*-value. Cytoscape software (version 3.9; Boston, MA, USA) was used to construct the ceRNA network, and a significance level of *p* < 0.05 was considered for each statistical test. GraphPad Prism (version 8.0; La Jolla, CA, USA) software was also used to draw the graphs.

## Results

### Identification of mRNAs and lncRNAs

**FIGURE 1:**
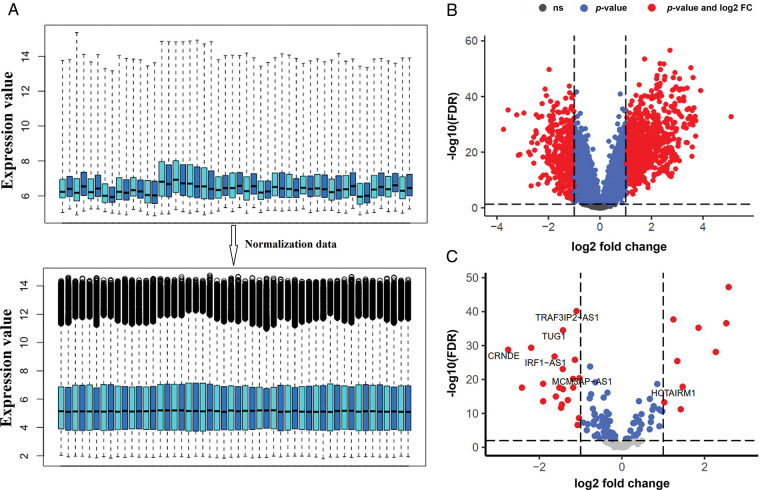
The expression of a large number of lncRNAs is altered in ALL. (A) Box plot of GSE67684 data normalization results. To reduce the dimensions only the number of samples is shown. (B) Differentially expressed mRNAs after normalization; |logFC| > 1 and FDR < 0.01. (C) Differentially expressed lncRNAs; (logFC; log2 fold change, FDR; false discover rate).

Genes with zero or near-zero expression were first excluded from further analysis. As a result, 9189 mRNAs and 423 lncRNAs with high probe intensities in ALL of the GSE67684 samples were identified. Then, the data normalization was performed as shown in Fig. 1A. Differential gene expression between day zero and day eight showed that 714 mRNAs increased and 379 mRNAs decreased; |logFC| > 1, and FDR < 0.05 (Fig. 1B). The full list of deregulated genes is summarized in “Suppl. Data 1”. On the other hand, 52 lncRNAs showed significant alterations in their expression. Twenty-one lncRNAs showed elevated expression on day eight compared to day zero samples, whereas 31 lncRNAs showed decreased expression (Fig. 1C, |logFC| > 1, FDR < 0.05).

### CeRNA network of lncRNAs involved in ALL development and related-pathway

**FIGURE 2:**
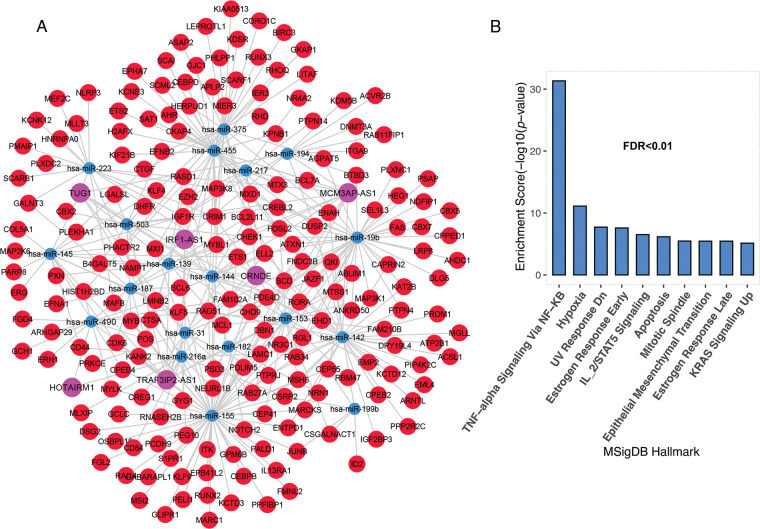
The lncRNAs; IRF1-AS1, MCM3AP-AS1, TRAF3IP2-AS1, HOTAIRM1, CRNDE, and TUG1 showed a high degree in the ceRNA network. (A) The ceRNA network of mRNAs and lncRNAs had significant alterations in ALL. (B) Enrichment results of genes presented in ceRNA network. The X-axis shows the GSEA pathways identified in the ceRNA network. The −log10 of the *p*-value is shown on Y-axis based on the enrichment results.

The most important function of lncRNAs is that they act as a sponge for miRNAs. The ceRNA network was constructed using lncRNAs and mRNAs identified in the previous step. The findings revealed that 10 of the 52 lncRNAs; C10ORF25, MIR22HG, IRF1-AS1, C4ORF46, MCM3AP-AS1, HAR1A, TRAF3IP2-AS1, HOTAIRM1, CRND1, CRNDE, and TUG1, were linked with 83 miRNAs based on miRcode data. The miRNA and mRNA interaction results were evaluated based on the Targetscan and miRTarBase databases. As a result, 19 miRNAs were identified as being associated with 356 common mRNAs in both databases. Unrelated candidates were excluded from the study process. The results from the previous processes were merged to create a ceRNA network, which was then used to identify significant lncRNAs. This network contained 6 lncRNA nodes, 17 miRNA nodes, 196 mRNA nodes, and 369 edges (Fig. 2A). The results showed that six lncRNAs, namely IRF1-AS1, MCM3AP-AS1, TRAF3IP2-AS1, HOTAIRM1, CRNDE, and TUG1, interact with many miRNAs targeting deregulated mRNAs in ALL (Fig. 2A and “Suppl. Data 2”). Enrichment results for the mRNAs in the ceRNA network showed that these are involved in inflammation through TNF-alpha, hypoxia, DNA repair (UV Response Dn), apoptosis, metastasis, proliferation (Mitotic Spindle), and other pathways related to mutation (Fig. 2B).

### Association of IRF1-AS1, MCM3AP-AS1, and TRAF3IP2-AS1 subnets to cancer progression pathways

**FIGURE 3:**
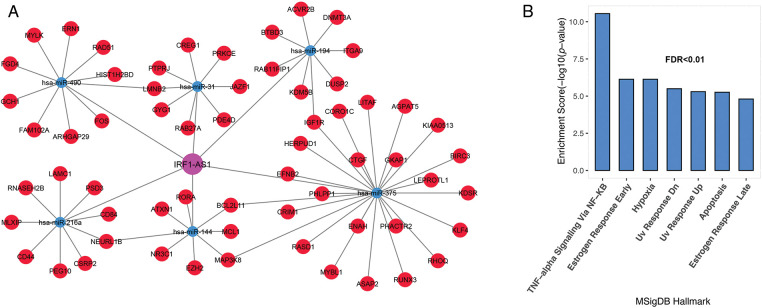
IRF1-AS1 subnet genes are involved in inflammation, DNA repair, and apoptotic pathways. (A) The subnet associated with IRF1-AS1-miRNA-mRNA is shown. (B) Enrichment results of genes involved in the IRF1-AS1-miRNA-mRNA axis in ALL. The X-axis shows the GSEA pathways identified in the IRF1-AS1 ceRNA network. The −log10 of the *p*-value is shown on Y-axis based on the enrichment results.

Previous studies have shown the association of HOTAIRM1, CRNDE, and TUG1 expression in the pathogenesis of various cancers, including ALL [20,21]. Hence, we decide to investigate the association of IRF1-AS1, MCM3AP-AS1, and TRAF3IP2-AS1 expression and their possible role in ALL, to which less attention has been paid. To better understand the biological functions of the three lncRNAs, subnetworks were built for each of them, and genes associated with each lncRNA were enriched. The results of the IRF1-AS1 associated subnet showed that genes connected to IRF1-AS1 are involved in pathways such as TNF-alpha signaling via NF-κB, apoptosis, DNA repair, and hypoxia (Figs. 3A and 3B, FDR < 0.01).

**FIGURE 4:**
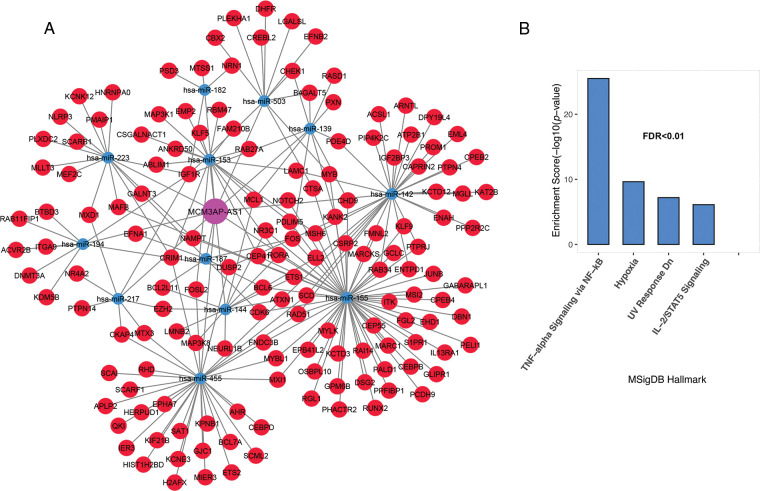
Inflammation and hypoxia are linked to the MCM3AP-AS1 subnet genes. (A) The subnet connected to MCM3AP-AS1 is shown. (B) Based on MsigDB data, enrichment results for MCM3AP-AS1-related genes are displayed. The X-axis shows the GSEA pathways identified in the MCM3AP-AS1 ceRNA network. The −log10 of the *p*-value is shown on Y-axis based on the enrichment results.

The MCM3AP-AS1 subnet enrichment was observed to be associated with genes related to TNF-alpha signaling via NF-κB, hypoxia, IL-2/STAT5 signaling, and DNA repair (Figs. 4A and 4B, FDR < 0.01). These results suggest that genes linked to each lncRNA are involved in vital biological processes associated with cancer cells. Therefore, they can play a vital role in the molecular pathogenesis of ALL.

**FIGURE 5:**
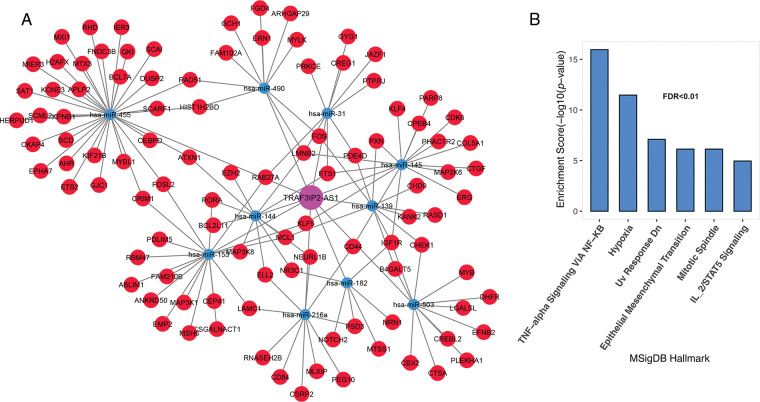
TRAF3IP2-AS1 subnet genes are involved in inflammation, proliferation, and metastasis. (A) The TRAF3IP2-AS1-related subnet. (B) Enrichment results for TRAF3IP2-AS1-related genes based on MsigDB data. The X-axis shows the GSEA pathways identified in the TRAF3IP2-AS1 ceRNA network. The −log10 of the *p*-value is shown on the Y-axis based on the enrichment results.

Genes associated with TRAF3IP2-AS1 are involved in TNF-alpha signaling through NF-κB, hypoxia, mitotic spindle, IL-2/STAT5 signaling, DNA repair, and epithelial-mesenchymal transition (Figs. 5A and 5B, FDR < 0.01).

### Expression changes of IRF1-AS1, MCM3AP-AS1, TRAF3IP2-AS1

**FIGURE 6:**
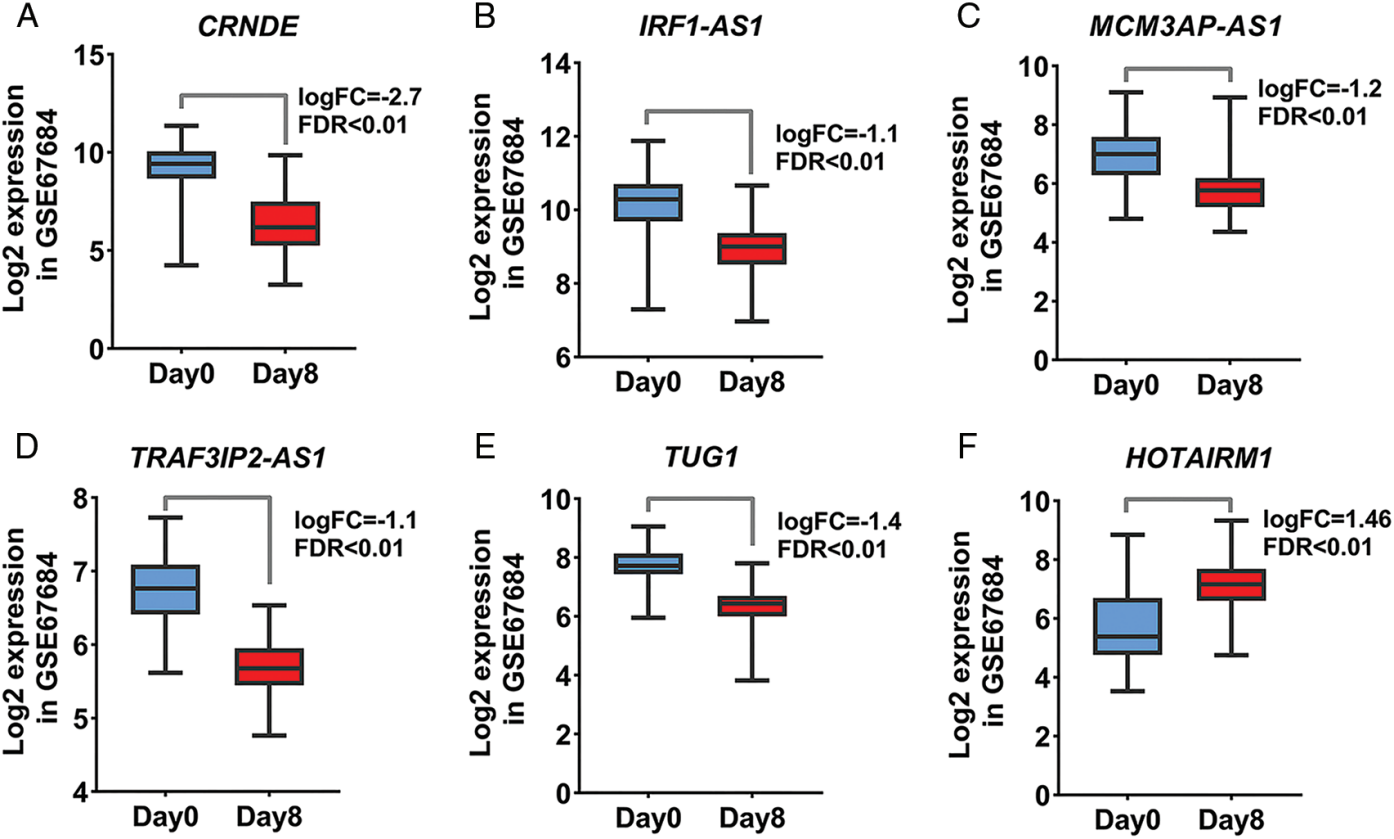
The expression of candidate lncRNA is significantly altered. (A–F) Changes in the expression of the identified lncRNA based on the normalized matrix of the GSE67684 study are shown. The graphs were created using normalized data on a logarithmic scale based on 2. The expression value of each gene in each sample was calculated based on samples on day zero and day eight of the studied dataset.

**FIGURE 7:**
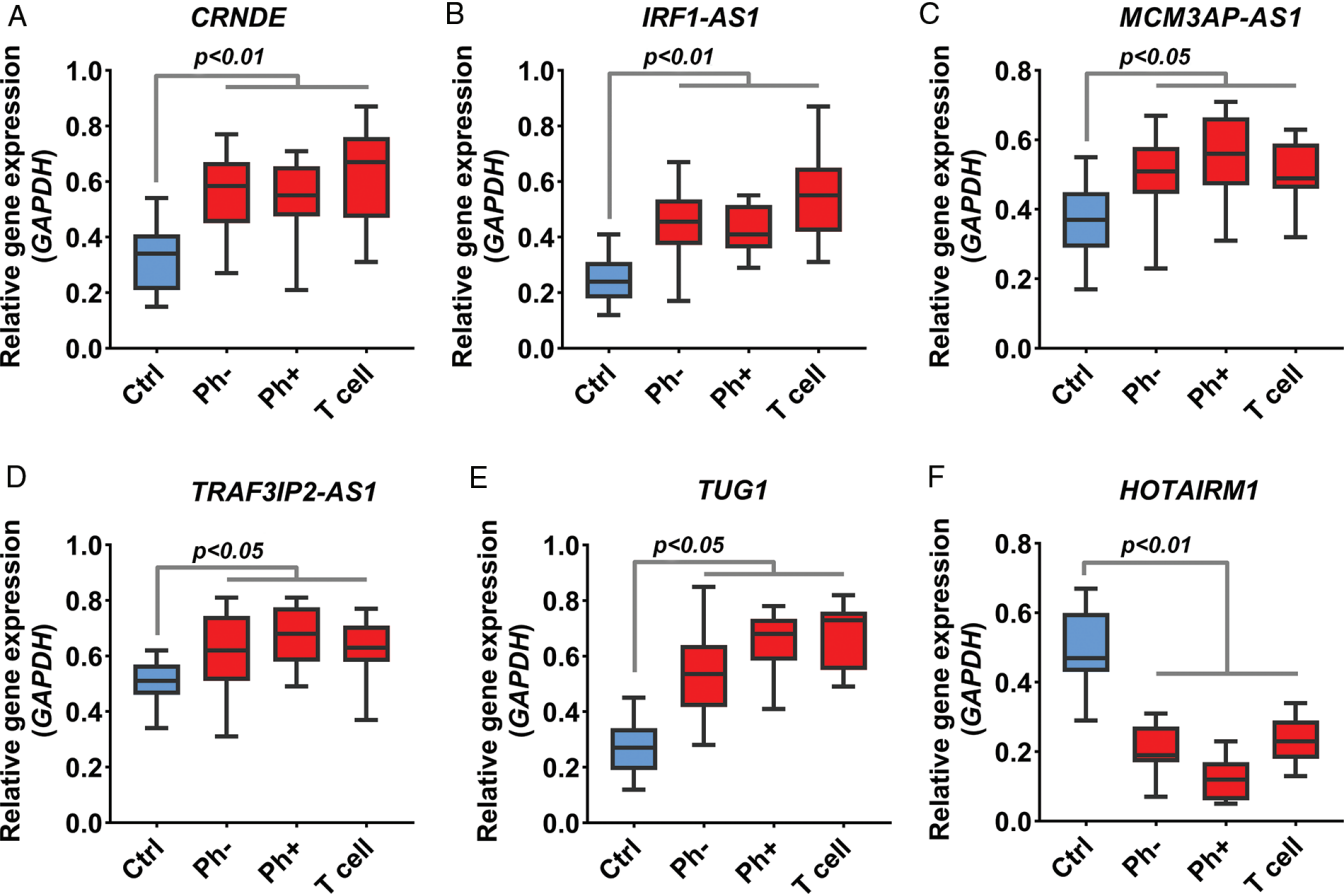
Expression change of IRF1-AS1, TRAF3IP2-AS1, and MCM3AP-AS1. (A–F) The expression level of candidate lncRNA in ALL samples compared to normal samples is shown based on RT-qPCR results. The expression level of each lncRNA in each sample was calculated based on 2^−▵Ct^. The expression value of each gene in each sample was calculated based on the average of three replicates.

Acute lymphoblastic leukemia patients and control samples were used to confirm the results obtained from the previous bioinformatics approach. As shown in Fig. 1, the expression of all candidate lncRNAs except HOTAIRM1, decreased on day eight compared to day zero based on GSE67684 (Fig. 6, FDR < 0.01). Using RT-qPCR, the expression of IRF1-AS1, MCM3AP-AS1, TRAF3IP2-AS1, CRNDE, and TUG1 was significantly higher in ph+, ph−, and T cell ALL subgroups compared to the control group (Figs. 7A–7E, *p* < 0.01). In contrast, HOTAIRM1 expression showed a significant decrease (Fig. 7F, *p* < 0.01).

These results indicate that the expression of IRF1-AS1, MCM3AP-AS1, TRAF3IP2-AS1, CRNDE, and TUG1 in ALL samples is significantly altered. Next, the relationship between the expression changes of lncRNAs and clinical pathological features was investigated. However, no significant relationship was found.

## Discussion

Different molecular studies have shown that lncRNAs can play a significant role in the pathogenesis of ALL through controlling many cellular processes [22]. Therefore, it is vital to identify lncRNAs involved in the development and progression of ALL. Given that, lncRNA inhibitors have entered the second phase of the clinical trial and have raised therapeutic hopes [23]. This study aimed to identify essential lncRNAs in ALL molecular pathogenesis as well as their mechanisms of action via the ceRNA network. For this purpose, using GEO data, we first identified lncRNAs and mRNAs whose expression changes dramatically during disease progression. Then, the associated ceRNA network and its signaling pathways were identified using bioinformatics tools. Finally, RT-qPCR was used to assess the expression of candidate lncRNAs in ALL samples.

The results of differential expression of lncRNAs in ALL samples showed that the expression of 52 lncRNAs changed significantly. Six lncRNAs were linked to many newly discovered miRNA-mRNA axes during ALL progression, including IRF1-AS1, MCM3AP-AS1, TRAF3IP2-AS1, HOTAIRM1, CRNDE, and TUG1. Therefore, it is suggested that these 6 lncRNAs can play a more prominent role in the development of ALL in the miRNA-mRNA-lncRNA axis. Additionally, the ceRNA network shows that these lncRNAs might have roles in the progression of ALL. According to the *in silico* study, except for HOTAIRM1, all showed decreasing expression levels on day eight compared to day zero. However, RT-qPCR data revealed the opposite. The most important reason for this divergence could be that the GSE67684 study employed an ALL-BFM treatment for patients during this period, which is an effective chemotherapy regimen in pediatric ALL patients and young adults [24,25]. Furthermore, the absence of normal subjects in the GSE67684 investigation prevented us from determining the trend of the discovered lncRNAs during the progression of ALL. A previous study has shown that HOTAIRM1 expression is increased in acute myeloid leukemia, and its expression level is associated with the prognosis of patients [26]. HOTAIRM 1 also has specific expression in blood cell precursor lines, and changes in its expression can affect apoptosis and cell proliferation in myeloid cell lines [27]. HOTAIRM1 regulates the activity of the WNT pathway [27], which plays a vital role in the development and malignancy of ALL [28]. On the other hand, it has been demonstrated that CRNDE expression is upregulated in ALL and that this upregulation can trigger apoptosis and inhibit the growth of ALL cell lines. Additionally, CRNDE can contribute to the development of ALL via the ceRNA network in the CREB protein axis [20]. Also, CRNDE promotes cancer cell proliferation, migration, and invasion and suppresses apoptosis through complicated mechanisms, that result in the initialization and development of human cancers [29]. TUG1 is significantly increased in ph-ALL compared to the control group, and its expression can be a potential prognostic biomarker in ALL [21]. It has been reported that TUG1 can activate the MAPK1/ERK pathway through the ceRNA network, which is crucial for the pathogenesis of acute myeloid leukemia [30]. Numerous studies show that TUG1 controls drug resistance, cell differentiation, invasion, metastasis, apoptosis, tumor growth, and cell metabolism in various types of cancer [31]. Studies in other cancers have shown that MCM3AP-AS1 can play an oncogenic role in liver, breast, colorectal, lung, and thyroid cancers [32–36]. Our *ex vivo* results also showed that the expression of IRF1-AS1, MCM3AP-AS1, TRAF3IP2-AS1, CRNDE, and TUG1 in ALL samples was significantly increased compared to the control group. HOTAIRM1 expression, on the other hand, decreased in ALL samples. We have shown for the first time that the expression levels of IRF1-AS1, MCM3AP-AS1, and TRAF3IP2-AS1 are significantly increased in ALL patients and the mentioned lncRNAs could be involved in the progression and malignancy of ALL. We, therefore, suggest that IRF1-AS1, MCM3AP-AS1, and TRAF3IP2-AS1 could be appropriate therapeutic and diagnostic targets in ALL.

The subnet analyses showed that IRF1-AS1, MCM3AP-AS1, and TRAF3IP2-AS1 are associated with genes involved in TNF-alpha signaling via NF-κB, hypoxia, mitotic spindle, IL-2/STAT5 signaling, DNA repair, and epithelial-mesenchymal transition. A previous study has illustrated that decreased expression of MCM3AP-AS1 in liver cancer cell lines can induce apoptosis through the ceRNA network [35]. In breast cancer, MCM3AP-AS1 has been reported to induce apoptosis through sponging microRNAs [32]. In addition, extensive studies have shown that the previously mentioned pathways play essential roles in the pathogenesis of ALL, and are among the main pathways of cancer cells [37–41]. These findings suggest that the expression changes of IRF1-AS1, MCM3AP-AS1, and TRAF3IP2-AS1 can play an oncogenic role in ALL. This oncogenic role is achieved through regulating TNF-alpha signaling via NF-κB, hypoxia, the mitotic spindle, IL-2/STAT5 signaling, DNA repair, and epithelial-mesenchymal transition pathways. The IRF1-AS1, MCM3AP-AS1, and TRAF3IP2-AS1 subnetworks also revealed that these lncRNAs could sponge hsa-miR-144, which has previously been shown to regulate cell proliferation and apoptosis in ALL cell lines [42].

On the other hand, studies have shown that hsa-miR-155 can regulate immune and inflammatory responses in ALL, and our results showed that this miRNA can interact with MCM3AP-AS1 and inflammation-related genes [43]. Interestingly, the ceRNA network of this study showed that hsa-miR-223 could interact with MCM3AP-AS1, and hsa-miR-22 target genes are involved in NF-κB pathways. It has been shown that this miRNA can regulate the NF-κB pathway in ALL [44].

One of the limitations of this study was the lack of access to data on days 15 and 30 in the GSE67684 study, which did not allow further investigation of the changes in the expression of this lncRNA after treatment on the above days. However, we could not conduct a more accurate analysis due to a lack of samples in various subtypes. It should also be noted that these results need to be further confirmed *in vitro* in other subtypes to find out how MCM3AP-AS1, TRAF3IP2-AS1, and IRF1-AS1 cause each ALL subtype at the molecular level.

## Conclusion

According to *in silico* and *ex vivo* studies, MCM3AP-AS1, TRAF3IP2-AS1, and IRF1-AS1 expression increased in ALL samples, implying that they may have oncogenic functions. Our findings further suggest that the lncRNAs indicated have a role in the pathogenesis of ALL by regulating pathways including TNF-alpha signaling via NF-κB, hypoxia, mitotic spindle, IL-2 and STAT5 signaling, DNA repair, and epithelial-mesenchymal transition via the ceRNA network. MCM3AP-AS1, TRAF3IP2-AS1, and IRF1-AS1 expression could be identified as potential therapeutic and diagnostic targets in ALL patients.

## Supplementary Materials

SUPPLEMENTAL DATA 1 List of differentially expressed LncRNAs identified in GSE67684.

SUPPLEMENTAL DATA 2 List of interactions between mRNA-miRNA-lncRNA.

## Data Availability

The processed raw data in this study is publicly available on GEO database (https://www.ncbi.nlm.nih.gov/geo/) with accession number of GSE67684. The datasets used and/or analyzed during the current study are available to the corresponding author upon request.

## References

[1] DeAngelo, D. J., Jabbour, E., Advani, A. (2020). Recent advances in managing acute lymphoblastic leukemia. American Society of Clinical Oncology Educational Book*,* 40 *(*40*),* 330–342. DOI 10.1200/EDBK_280175. 32421447

[2] Pui, C. H., Relling, M. V., Downing, J. R. (2004). Acute lymphoblastic leukemia. New England Journal of Medicine*,* 350 *(*15*),* 1535–1548. DOI 10.1056/NEJMra023001. 15071128

[3] Roberts, K. G., Li, Y., Payne-Turner, D., Harvey, R. C., Yang, Y. L. et al. (2014). Targetable kinase-activating lesions in Ph-like acute lymphoblastic leukemia. New England Journal of Medicine*,* 371 *(*11*),* 1005–1015. DOI 10.1056/NEJMoa1403088. 25207766 PMC4191900

[4] Ferrando, A. A., Look, A. T. (2003). Gene expression profiling in T-cell acute lymphoblastic leukemia. Seminars in Hematology*,* 40 *(*4*),* 274–280. DOI 10.1016/S0037-1963(03)00195-1. 14582078

[5] Figueroa, M. E., Chen, S. C., Andersson, A. K., Phillips, L. A., Li, Y. et al. (2013). Integrated genetic and epigenetic analysis of childhood acute lymphoblastic leukemia. The Journal of Clinical Investigation*,* 123 *(*7*),* 3099–3111. DOI 10.1172/JCI66203. 23921123 PMC3696550

[6] Mahdevar, M., Vatandoost, J., Forootan, F. S., Kiani-Esfahani, A., Esmaeili, M. et al. (2021). Steroid receptor RNA activator gene footprint in the progression and drug resistance of colorectal cancer through oxidative phosphorylation pathway. Life Sciences*,* 285 *(*3*),* 119950. DOI 10.1016/j.lfs.2021.119950. 34520769

[7] Zhang, R., Xia, L. Q., Lu, W. W., Zhang, J., Zhu, J. S. (2016). LncRNAs and cancer. Oncology Letters*,* 12 *(*2*),* 1233–1239. DOI 10.3892/ol.2016.4770. 27446422 PMC4950797

[8] Cuadros, M., Andrades, Á., Coira, I. F., Baliñas, C., Rodríguez, M. I. et al. (2019). Expression of the long non-coding RNA TCL6 is associated with clinical outcome in pediatric B-cell acute lymphoblastic leukemia. Blood Cancer Journal*,* 9 *(*12*),* 1–5. DOI 10.1038/s41408-019-0258-9. PMC6877621 31767830

[9] Huang, X., Huang, L., Xie, Q., Zhang, L., Huang, S. et al. (2021). LncRNAs serve as novel biomarkers for diagnosis and prognosis of childhood ALL. Biomarker Research*,* 9 *(*1*),* 1–11. DOI 10.1186/s40364-021-00303-x. 34112247 PMC8193891

[10] Gao, J., Wang, F., Wu, P., Chen, Y., Jia, Y. (2020). Aberrant LncRNA expression in leukemia. Journal of Cancer*,* 11 *(*14*),* 4284–4296. DOI 10.7150/jca.42093. 32368311 PMC7196264

[11] Wang, W., Lyu, C., Wang, F., Wang, C., Wu, F. et al. (2021). Identification of potential signatures and their functions for acute lymphoblastic leukemia: a study based on The Cancer Genome Atlas. Frontiers in Genetics*,* 12*,* 930. DOI 10.3389/fgene.2021.656042. PMC8290159 34295352

[12] Xiao, S., Xu, N., Ding, Q., Huang, S., Zha, Y. et al. (2020). LncRNA VPS9D1-AS1 promotes cell proliferation in acute lymphoblastic leukemia through modulating GPX1 expression by miR-491-5p and miR-214-3p evasion. Bioscience Reports*,* 40 *(*10*),* BSR20193461. DOI 10.1042/BSR20193461. 32808668 PMC7536331

[13] Harrison, A. P., Johnston, C. E., Orengo, C. A. (2007). Establishing a major cause of discrepancy in the calibration of Affymetrix GeneChips. BMC Bioinformatics*,* 8 *(*1*),* 1–11. DOI 10.1186/1471-2105-8-195. 17562008 PMC1904248

[14] Shakya, K., Ruskin, H., Kerr, G., Crane, M., Becker, J. (2010). Comparison of microarray preprocessing methods. In: Advances in computational biology, vol. 680, pp. 139–147. Springer. 10.1007/978-1-4419-5913-3_16 20865495

[15] Durinck, S., Moreau, Y., Kasprzyk, A., Davis, S., de Moor, B. et al. (2005). BioMart and bioconductor: A powerful link between biological databases and microarray data analysis. Bioinformatics*,* 21 *(*16*),* 3439–3440. DOI 10.1093/bioinformatics/bti525. 16082012

[16] Jeggari, A., Marks, D. S., Larsson, E. (2012). miRcode: A map of putative microRNA target sites in the long non-coding transcriptome. Bioinformatics*,* 28 *(*15*),* 2062–2063. DOI 10.1093/bioinformatics/bts344. 22718787 PMC3400968

[17] Shannon, P., Markiel, A., Ozier, O., Baliga, N. S., Wang, J. T. et al. (2003). Cytoscape: A software environment for integrated models of biomolecular interaction networks. Genome Research*,* 13 *(*11*),* 2498–2504. DOI 10.1101/gr.1239303. 14597658 PMC403769

[18] Maere, S., Heymans, K., Kuiper, M. (2005). *BiNGO*: A Cytoscape plugin to assess overrepresentation of gene ontology categories in biological networks. Bioinformatics*,* 21 *(*16*),* 3448–3449. DOI 10.1093/bioinformatics/bti551. 15972284

[19] Schmittgen, T. D., Livak, K. J. (2008). Analyzing real-time PCR data by the comparative CT method. Nature Protocols*,* 3 *(*6*),* 1101–1108. DOI 10.1038/nprot.2008.73. 18546601

[20] Wang, W., Wu, F., Ma, P., Gan, S., Li, X. et al. (2020). LncRNA CRNDE promotes the progression of B-cell precursor acute lymphoblastic leukemia by targeting the miR-345-5p/CREB axi. Molecules and Cells*,* 43*,* 718–727. DOI 10.14348/molcells.2020.0065. 32868489 PMC7468588

[21] Zeng, P., Chai, Y., You, C., Yue, L., Wu, C. et al. (2021). Correlation analysis of long non-coding RNA TUG1 with disease risk, clinical characteristics, treatment response, and survival profiles of adult Ph^−^ Acute lymphoblastic leukemia. Journal of Clinical Laboratory Analysis*,* 35 *(*8*),* e23583. DOI 10.1002/jcla.23583. 34251066 PMC8373340

[22] Illarregi, U., Telleria, J., Bilbao‐Aldaiturriaga, N., Lopez‐Lopez, E., Ballesteros, J. et al (2022). lncRNA deregulation in childhood acute lymphoblastic leukemia: A systematic review. International Journal of Oncology*,* 60 *(*5*),* 1–9. DOI 10.3892/ijo.2022.5348. 35419612

[23] Zhou, T., Kim, Y., MacLeod, A. R. (2016). Targeting long noncoding RNA with antisense oligonucleotide technology as cancer therapeutics. In: Long Non-Coding RNAs, vol. 1402, pp. 199–213. Springer. 10.1007/978-1-4939-3378-5_16 26721493

[24] Schrappe, M., Reiter, A., Ludwig, W. D., Harbott, J., Zimmermann, M. et al. (2000). Improved outcome in childhood acute lymphoblastic leukemia despite reduced use of anthracyclines and cranial radiotherapy: results of trial ALL-BFM 90. Blood, 95 (11)*,* 3310–3322. DOI 10.1182/blood.V95.11.3310. 10828010

[25] Gaynon, P., Bleyer, W. A., Albo, V., Grossman, N., Novak, L. T. et al. (1988). Intensive therapy for children with acute lymphoblastic leukaemia and unfavourable presenting features: Early conclusions of study CCG-106 by the Childrens Cancer Study Group. The Lancet*,* 332 *(*8617*),* 921–924. DOI 10.1016/S0140-6736(88)92596-2. 2902379

[26] Díaz-Beyá, M., Brunet, S., Nomdedéu, J., Pratcorona, M., Cordeiro, A. et al. (2015). The lincRNA HOTAIRM1, located in the HOXA genomic region, is expressed in acute myeloid leukemia, impacts prognosis in patients in the intermediate-risk cytogenetic category, and is associated with a distinctive microRNA signature. Oncotarget*,* 6 *(*31*),* 31613–31627. DOI 10.18632/oncotarget.5148. 26436590 PMC4741628

[27] Chen, L., Hu, N., Wang, C., Zhao, H. (2020). HOTAIRM1 knockdown enhances cytarabine-induced cytotoxicity by suppression of glycolysis through the Wnt/β-catenin/PFKP pathway in acute myeloid leukemia cells. Archives of Biochemistry and Biophysics*,* 680*,* 108244. DOI 10.1016/j.abb.2019.108244. 31904363

[28] Weerkamp, F., van Dongen, J., Staal, F. (2006). Notch and Wnt signaling in T-lymphocyte development and acute lymphoblastic leukemia. Leukemia*,* 20 *(*7*),* 1197–1205. DOI 10.1038/sj.leu.2404255. 16688226

[29] Zhang, J., Yin, M., Peng, G., Zhao, Y. (2018). CRNDE: An important oncogenic long non-coding RNA in human cancers. Cell Proliferation*,* 51 *(*3*),* e12440. DOI 10.1111/cpr.12440. 29405523 PMC6528921

[30] Li, G., Zheng, P., Wang, H., Ai, Y., Mao, X. (2019). Long non-coding RNA TUG1 modulates proliferation, migration, and invasion of acute myeloid leukemia cells via regulating miR-370-3p/MAPK1/ERK. OncoTargets and Therapy*,* 12*,* 10375. DOI 10.2147/OTT. 31819520 PMC6890183

[31] Zhou, H., Sun, L., Wan, F. (2019). Molecular mechanisms of TUG1 in the proliferation, apoptosis, migration and invasion of cancer cells. Oncology Letters*,* 18 *(*5*),* 4393–4402. DOI 10.3892/ol.2019.10848. 31611948 PMC6781668

[32] Chen, Q., Xu, H., Zhu, J., Feng, K., Hu, C. (2020). LncRNA MCM3AP-AS1 promotes breast cancer progression via modulating miR-28-5p/CENPF axis. Biomedicine & Pharmacotherapy*,* 128 *(*6*),* 110289. DOI 10.1016/j.biopha.2020.110289. 32485570

[33] Li, X., Yu, M., Yang, C. (2020). YY1-mediated overexpression of long noncoding RNA MCM3AP-AS1 accelerates angiogenesis and progression in lung cancer by targeting miR-340-5p/KPNA4 axis. Journal of Cellular Biochemistry*,* 121 *(*3*),* 2258–2267. DOI 10.1002/jcb.29448. 31693222

[34] Liang, M., Jia, J., Chen, L., Wei, B., Guan, Q. et al. (2019). LncRNA MCM3AP-AS1 promotes proliferation and invasion through regulating miR-211-5p/SPARC axis in papillary thyroid cancer. Endocrine*,* 65 *(*2*),* 318–326. DOI 10.1007/s12020-019-01939-4. 31030335

[35] Wang, Y., Yang, L., Chen, T., Liu, X., Guo, Y. et al. (2019). A novel lncRNA MCM3AP-AS1 promotes the growth of hepatocellular carcinoma by targeting miR-194-5p/FOXA1 axis. Molecular Cancer*,* 18 *(*1*),* 1–16. DOI 10.1186/s12943-019-0957-7. 30782188 PMC6381672

[36] Zhou, M., Bian, Z., Liu, B., Zhang, Y., Cao, Y. et al. (2021). Long noncoding RNA MCM3AP-AS1 enhances cell proliferation and metastasis in colorectal cancer by regulating miR-193a-5p/SENP1. Cancer Medicine*,* 10 *(*7*),* 2470–2481. DOI 10.1002/cam4.3830. 33686713 PMC7982620

[37] Bloehdorn, J., Braun, A., Taylor-Weiner, A., Jebaraj, B. M. C., Robrecht, S. et al. (2021). Multi-platform profiling characterizes molecular subgroups and resistance networks in chronic lymphocytic leukemia. Nature Communications*,* 12 *(*1*),* 1–18. DOI 10.1038/s41467-021-25403-y. PMC8438057 34518531

[38] Ganster, C., Neesen, J., Zehetmayer, S., Jäger, U., Esterbauer, H. et al. (2009). DNA repair polymorphisms associated with cytogenetic subgroups in B-cell chronic lymphocytic leukemia. Genes, Chromosomes and Cancer*,* 48 *(*9*),* 760–767. DOI 10.1002/gcc.20680. 19484764

[39] Konopleva, M., Benito, J., Shi, Y. X., Konoplev, S., Kornblau, S. M. et al. (2009). Therapeutic targeting of the hypoxic microenvironment in acute lymphocytic leukemia. Blood*,* 114 *(*22*),* 2040. DOI 10.1182/blood.V114.22.2040.2040.

[40] Mezzatesta, C., Bornhauser, B. C. (2019). Exploiting necroptosis for therapy of acute lymphoblastic leukemia. Frontiers in Cell and Developmental Biology*,* 7*,* 40. DOI 10.3389/fcell.2019.00040. 30941349 PMC6433701

[41] Oliveira, M. L., Akkapeddi, P., Ribeiro, D., Melão, A., Barata, J. T. (2019). IL-7R-mediated signaling in T-cell acute lymphoblastic leukemia: An update. Advances in Biological Regulation*,* 71 *(*33*),* 88–96. DOI 10.1016/j.jbior.2018.09.012. 30249539 PMC6386770

[42] Jin, J., Wang, Y., Xu, Y., Zhou, X., Liu, Y. et al. (2017). MicroRNA-144 regulates cancer cell proliferation and cell-cycle transition in acute lymphoblastic leukemia through the interaction of FMN2. The Journal of Gene Medicine*,* 19 *(*6–7*),* e2898. DOI 10.1002/jgm.2898. 27556228

[43] Hassan, S. S., El-Khazragy, N., Elshimy, A. A., Aboelhussein, M. M., Saleh, S. A. et al. (2020). *In vitro* knock-out of miR-155 suppresses leukemic and HCV virus loads in pediatric HCV-4-associated acute lymphoid leukemia: A promising target therapy. Journal of Cellular Biochemistry*,* 121 *(*4*),* 2811–2817. DOI 10.1002/jcb.29512. 31696995

[44] Kumar, V., Palermo, R., Talora, C., Campese, A., Checquolo, S. et al. (2014). Notch and NF-kB signaling pathways regulate miR-223/FBXW7 axis in T-cell acute lymphoblastic leukemia. Leukemia*,* 28 *(*12*),* 2324–2335. DOI 10.1038/leu.2014.133. 24727676

